# Original compound with anticonvulsant activity in the treatment of alcoholism

**DOI:** 10.1192/j.eurpsy.2021.2171

**Published:** 2021-08-13

**Authors:** E. Markova, I. Savkin, M. Knyazheva, T. Shushpanova

**Affiliations:** 1 Neuroimmunology Lab, State Research Institute of Fundamental and Clinical Immunology, Novosibirsk, Russian Federation; 2 Clinical Psychoneuroimmunology And Neurobiology Lab, Mental Health Research Institute, Tomsk, Russian Federation

**Keywords:** Original anticonvulsant, alcoholism

## Abstract

**Introduction:**

Original compound ortho-fluorobenzonal, a barbiturate derivative, is shown to reveal strong anticonvulsant activity by means increasing GABA-mediation. Disturbance of GABA(A) -receptors functions play an essential role in both alcoholism and epilepsy pathogenesis.

**Objectives:**

Taking into account the presence of GABA(A)-receptors on the lymphocytes surface and involvement of immune system in alcoholism pathogenesis, we investigated ortho-fluorobenzonal effects on the immune and nervous systems functional activities in mice with chronic alcohol exposure to find new perspective pharmacological substances in the treatment of alcoholism.

**Methods:**

(CBAxC57Bl/6) F1 male mice with 6-month 10% ethanol exposure were undergoing intragastric administration of original compound ortho-fluorobenzonal for 10 days. Animal’s alcohol consumption, behavior and immune parameters were estimated.

**Results:**

It was found that ethanol daily consumption decreased sharply starting from 2 days of ortho-fluorobenzonal administration and led to the cessation of ethanol consumption by the 4 day in mice with chronic alcohol exposure. Pronounced changes in motor and exploratory activities in “open field” test was registered in long-term alcoholized mice after 10- day course ortho-fluorobenzonal administration. The above behavioral changes were recorded against the brain cytokines synthesis modulation. We have shown also the modulation by ortho-fluorobenzonal of immune system functional activity, in particular, significant cellular and humoral immune response stimulation, estimated by the relative number of antibody forming cells and reaction of delayed-type hypersensitivity respectively.

**Conclusions:**

Original compound ortho-fluorobenzonal has a positive neuroimmunomodulation effect that manifests itself in the correction of iimmune and behavior disorders caused by the chronic ethanol exposure, therefore, this compound is promising in the therapy of alcoholism

**Disclosure:**

No significant relationships.

